# Fine-Mapping-Based Variant Prioritization and Genomic Prediction Enhance Genetic Analyses of Teat Traits in Pigs

**DOI:** 10.3390/ani16121855

**Published:** 2026-06-16

**Authors:** Dongbin Yao, Cai-Xia Yang, Bing Deng, Pan Wang, Shuaipeng He, Zhi-Qiang Du, Zuhong Liu

**Affiliations:** 1 College of Animal Science and Technology, Yangtze University, Jingzhou 434025, China; yaodb125@163.com (D.Y.); caixiayang@yangtzeu.edu.cn (C.-X.Y.); 2 Institute of Animal Husbandry and Veterinary Medicine, Wuhan Academy of Agricultural Sciences, Wuhan 430070, China; dengbing@wuhanagri.com; 3 Yangxin County Animal Breeding Farm, Yangxin, Huangshi 435200, China; 15002736993@163.com; 4 Laboratory Animal Center, Huazhong Agricultural University, Wuhan 430070, China; shuaipeng.he@mail.hzau.edu.cn

**Keywords:** GWAS, fine-mapping, genomic prediction, variant prioritization, pig teat traits

## Abstract

Identifying causal genetic variants and candidate genes underlying complex traits remains a central challenge in animal breeding and genetics. Genome-wide association studies (GWAS) are widely used for this purpose. However, their reliance on marginal variant effects and sensitivity to linkage disequilibrium (LD) can lead to redundant and less accurate identification of variants or genes of biological relevance. Here, we propose SNP prioritization (GWAS-based and fine-mapping-based) strategies within a unified framework, designed to improve the selection of more informative variants and candidate genes by explicitly modeling LD structure and genetic architectures of three pig teat-related traits (total teat number, teat symmetry, and teat adequacy). While GWAS prioritization favored variants with strong marginal effects, fine-mapping substantially improved joint explanatory performance and prediction accuracy. For total teat number, the best-performing fine-mapping-derived SNP subset achieved a mean PCC of 0.6599 across 10-fold cross-validation, compared with 0.3755 for GWAS-based prioritization. Similarly, for teat adequacy, the highest mean AUC increased from 0.7012 (GWAS) to 0.8547 (fine-mapping). Moreover, fine-mapping-derived SNP sets identified more coherent and trait-specific biological pathways and functionally relevant candidate genes. Taken together, our findings demonstrate that fine-mapping provides a more accurate and biologically meaningful framework for SNP and candidate gene prioritization, supporting its integration into genetic analysis and breeding applications.

## Simple Summary

Identifying causal genetic variants or genes underlying important traits in livestock is essential for better selection and breeding outcomes. A common strategy is to select variants based on their statistical significance in association studies. However, this approach can be misleading because nearby variants often carry confounding information due to linkage disequilibrium and may block true biological effects. Here, we compared the ability of traditional methods and a more advanced approach to identify informative genetic variants and candidate genes. Using three teat-related traits in pigs—total teat number, teat symmetry, and teat adequacy—we show that the advanced method effectively reduced redundancy, improved prediction accuracy, and identified biologically relevant genes and pathways. This suggests that optimization in the selection of genetic variants can lead to more accurate identification of functional genes, supporting better breeding decisions and livestock production.

## 1. Introduction

Genetic dissection and identification of causal variants underlying complex traits remain a major objective in animal breeding and genetics, with important implications for genomic selection and breeding efficiency [1,2]. Genome-wide association studies (GWAS) have become one of the most widely used approaches for detecting trait-associated variants and have been widely applied across livestock species to understand the genetic bases of economically important traits [3].

However, GWAS-based SNP prioritization is inherently limited by its reliance on marginal effect estimation and its sensitivity to linkage disequilibrium (LD) [4]. In regions of strong LD, variants often exhibit similar levels of statistical significance (collinearity), leading to variant redundancy and complicating the identification of truly informative or causal variants [5,6]. As a result, SNP sets derived from conventional significance thresholds may not accurately represent the underlying genetic architecture, potentially limiting their effectiveness in downstream applications, such as genomic prediction and biological interpretation [7].

To disentangle correlated association signals within genomic regions, fine-mapping approaches have been developed by explicitly modeling LD structure and allowing for multiple causal variants [8,9]. Compared to traditional significance-based ranking, Bayesian fine-mapping methods, such as SuSiE [10], estimate posterior inclusion probabilities (PIPs) that quantify the likelihood of each variant being causal, thereby providing a probabilistic framework for SNP prioritization [11,12]. While previous studies demonstrated the advantages of fine-mapping in improving mapping resolution and reducing LD-driven false prioritization [11,12], most focused on statistical properties, such as credible set size or localization accuracy [13]. Less attention has been given to how different SNP prioritization strategies influenced downstream analytical outcomes, particularly in terms of explanatory power, genomic prediction performance, and biological interpretability.

Teat-related traits in pigs, including total teat number, teat symmetry, and teat adequacy, are associated with reproductive performance and piglet survival [14,15,16,17]. Moreover, these traits are of varying genetic architectures, ranging from highly polygenic backgrounds to weak or diffuse effects, as demonstrated by GWAS results in a number of different pig breeds [18,19,20]. Therefore, pig teat traits can be used as a suitable and biologically relevant example for evaluating SNP prioritization strategies and their impact on the identification of candidate variants and genes.

Taiwanese Duroc pigs represent an important terminal sire line in commercial pig breeding programs and have been subjected to long-term artificial selection for growth, carcass, and reproductive performance. The relatively well-managed population structure and extensive breeding records make this population a valuable resource for investigating the genetic architecture of economically important traits and evaluating genomic analysis strategies.

In the present study, we established a unified analytical framework to systematically compare SNP prioritization strategies, based on GWAS significance and fine-mapping-derived PIPs. Specifically, we evaluated their performance across three complementary dimensions: (i) single-SNP and multi-SNP explanatory powers, (ii) genomic prediction accuracy using GBLUP-based models, and (iii) functional annotation and enrichment analyses to assess biological relevance. Our results show that fine-mapping-based prioritization, by explicitly accounting for LD structure and multi-signal architectures, offers a more efficient and biologically meaningful representation of genetic variants compared to conventional GWAS-based selection.

## 2. Materials and Methods

### 2.1. Animals

#### 2.1.1. Pig Population

From November 2022 to October 2024, a total of 771 pigs from a closed nucleus herd of Taiwanese Duroc origin were collected from a large commercial breeding farm in central China. The herd had been maintained as a closed breeding population for approximately three generations. The population consisted of 451 parental individuals and 320 offspring, of which 588 were boars and 183 were sows. Animals used in this study were randomly selected from the available breeding population, and no additional selection criteria were applied.

#### 2.1.2. Phenotype

At birth, sex and teat-related traits were recorded for all piglets. Teat traits included total teat number (TTN), teat symmetry (TS), and teat adequacy (TA). TS was defined as the symmetry between the left and right sides, where 0 indicated symmetry and 1 indicated asymmetry. TA was defined based on teat quantity (0 indicated acceptable, and 1 indicated unacceptable), i.e., pigs with at least six pairs of teats were considered acceptable according to Duroc breeding standards. Descriptive statistics of teat-related traits showed that TTN exhibited considerable phenotypic variation, ranging from 8 to 15 teats (Table A1). For the binary traits, TS and TA showed relatively balanced distributions between categories.

#### 2.1.3. DNA Extraction, Sequencing, and Quality Control

Genomic DNA was extracted from ear tissue using the phenol–chloroform method. DNA integrity was assessed by 0.8% agarose gel electrophoresis, and DNA concentration and purity were measured using a NanoDrop 2000 spectrophotometer (Thermo Fisher Scientific, Waltham, MA, USA), ensuring an OD260/280 ratio between 1.7 and 2.1 and a concentration above 50 ng/μL.

DNA samples from the 451 parental animals were sequenced at approximately 10× depth using the BGISEQ-500 platform (BGI, Wuhan, China) to generate whole-genome sequencing (WGS) data. After alignment to the Sus scrofa 11.1 reference genome, a total of 9,600,601 autosomal SNPs were identified. The 320 offspring individuals were genotyped using the Porcine 80 K SNP Array (Wuhan GeneTech Co., Ltd., Wuhan, China).

### 2.2. Genotype Quality Control and Population Structure

To ensure the reliability of downstream analyses, stringent quality control (QC) procedures were applied to the WGS data using PLINK (v1.90b6.21) [21,22]. Samples with a genotype missing rate greater than 2% (--mind 0.02) were excluded [21,23]. SNPs were filtered based on genotype missingness (--geno 0.05), minor allele frequency (--maf 0.01), and Hardy–Weinberg equilibrium (--hwe 1 × 10^−6^). Sample identifiers were standardized across genotype, phenotype, and covariate datasets prior to downstream analyses to ensure consistent sample matching [23].

LD-based pruning was performed using a sliding window approach (--indep-pairwise 50 5 0.2) [24]. Population structure was subsequently assessed using principal component analysis (PCA), and the top 10 principal components were computed [24,25]. Outlier individuals were identified based on a Z-score threshold of 3 using the leading principal components. Individuals located beyond three standard deviations from the main population cluster were considered potential population outliers and removed to minimize the influence of population stratification and ensure population homogeneity [21,25]. As a result, one outlier individual was excluded from all downstream genotype, phenotype, and covariate analyses to ensure dataset consistency, and the final dataset consisted of 770 individuals.

### 2.3. Construction of Reference Haplotype Panel

A high-quality reference haplotype panel was constructed from the filtered WGS dataset (*n* = 450) [22,26]. Genotype data were converted into Variant Call Format (VCF) using PLINK (v1.90b6.21) and subjected to a series of standardization procedures. Variant normalization, reference allele verification, and allele consistency checking were performed using bcftools (v1.16) against the Sscrofa11.1 reference genome [22,26]. Allele consistency checking was conducted by comparing chromosome positions, reference alleles, and alternative alleles with the reference genome to ensure uniform variant representation across the dataset. Strand alignment was performed to correct strand orientation discrepancies when necessary. Only biallelic single-nucleotide polymorphisms (SNPs) were retained, and ambiguous SNPs (A/T and C/G) were excluded to avoid strand inconsistency and potential allele assignment errors [21]. Haplotype phasing was subsequently performed using Beagle (v5.2) with default parameters to infer the underlying haplotype structure and generate phased haplotypes for the construction of the reference panel [27,28]. The resulting phased dataset was used as the reference panel for genotype imputation.

### 2.4. Chip Genotype Processing

Chip-based genotype data were processed independently and converted into VCF format using PLINK (v1.90b6.21). To ensure compatibility with the reference panel, variant positions and alleles were aligned between the chip dataset and the reference dataset [22,26]. Shared variants between the two datasets were first identified according to chromosome and physical position. Allele harmonization was then performed by comparing reference and alternative alleles between datasets to ensure consistent strand orientation and allele coding across datasets [22,29]. Variants with inconsistent allele coding were corrected by strand flipping when appropriate. Ambiguous SNPs (A/T and C/G) and variants with unresolved allele inconsistencies were excluded from subsequent analyses [22,29]. This step ensured accurate matching of variants for downstream imputation. Due to differences in variant representation and quality filtering between datasets, only variants with matching genomic positions and allele definitions were retained as anchor markers for imputation [22,26].

### 2.5. Genotype Imputation and Quality Control

Genotype imputation was performed using Beagle (v5.2) with the phased WGS reference panel constructed from 450 sequenced Taiwanese Duroc pigs [26,27]. The effective population size parameter was set to ne = 2000, and 12 computational threads were used. All remaining parameters were left at their default settings. The imputation process leveraged local LD patterns to infer unobserved genotypes in the chip dataset [22,29]. Post-imputation quality control was conducted using the dosage R-squared metric (DR^2^) reported by Beagle, which estimates the squared correlation between imputed genotype dosages and the corresponding true underlying genotypes and is widely used as a measure of imputation reliability [26,27]. Variants with DR^2^ > 0.8 were considered high-confidence imputed SNPs according to commonly used imputation quality standards and were retained for downstream analyses. Only variants with DR^2^ > 0.8 were retained as high-confidence imputed SNPs for downstream analyses. The filtered imputed dataset was converted into PLINK binary format using PLINK and further harmonized with the reference dataset by retaining common variants [23,30], resulting in a final high-density genotype dataset for downstream GWAS and fine-mapping analyses. After genotype imputation and quality control, the imputed chip dataset was merged with the WGS reference dataset using the common high-confidence SNP set. The resulting combined dataset comprised 770 individuals and 4,443,258 SNPs and was used for all downstream GWAS and fine-mapping analyses.

### 2.6. GWAS and LD Structure

To generate input statistics for fine-mapping analysis, GWAS and LD structure estimation were performed in the training dataset [31]. GWAS summary statistics and corresponding LD matrices were jointly used as input for downstream fine-mapping analysis, ensuring a consistent analytical framework across traits [32,33].

GWAS analyses were conducted using PLINK (v1.90b6.21) [23]. For continuous traits, single-marker linear regression was performed using the linear model, while for binary traits, logistic regression was applied using the logistic model, both including sex as a covariate.

The resulting SNP effect estimates (BETA) and their standard errors (SEs) were used to compute Z-scores [34,35], defined as the ratio of effect size to its standard error. This transformation enabled the integration of association signals across different traits and models into a unified statistical representation. In parallel, LD matrices were calculated using the same set of individuals to ensure consistency between summary statistics and LD structure. These matrices were used to capture the correlation structure among SNPs and served as covariance input for downstream fine-mapping analyses. For LD-based preprocessing, SNP clustering was performed using a 500 kb window and an r^2^ threshold of 0.8.

### 2.7. GWAS-Guided LD-Based SNP Selection Strategy

To improve the efficiency and interpretability of downstream fine-mapping analyses, we adopted a GWAS-guided LD-based SNP selection strategy to preprocess genome-wide variants [1,7,36]. By jointly considering genomic correlation structure and association significance, this strategy improved the stability and interpretability of fine-mapping results while substantially reducing computational burden [9,37,38].

For high-density genotype datasets, LD-based clumping was performed using PLINK (v1.90b6.21) [7,23,30]. GWAS summary statistics were used as input, and SNPs were grouped into LD clusters within a 500 kb window based on an r^2^ threshold of 0.8. Each LD cluster consisted of a lead SNP and a set of correlated variants, as recorded in the resulting clumped file. Since LD structure is determined solely by genotype data and is independent of specific traits, LD clustering was performed once per species and reused across all traits within the same population [5,7].

Based on the predefined LD clusters, trait-specific representative SNP selection was conducted [1,4]. Within each LD cluster, the SNP with the smallest GWAS *p*-value for the corresponding trait was selected as the representative variant. This procedure was implemented using custom Python scripts, ensuring that only one SNP per LD cluster was retained while removing redundant signals.

As a result, a reduced SNP set with low LD redundancy and enriched phenotypic association signals was generated for each trait. These SNP sets were subsequently used as input for chromosome-wise fine-mapping analyses using Sum of Single Effects Regression using Summary Statistics (SuSiE-RSS), and the results were aggregated across chromosomes to obtain genome-wide posterior inclusion probabilities for each variant.

### 2.8. Fine-Mapping Using SuSiE-RSS

Fine-mapping was performed using SuSiE-RSS based on GWAS summary statistics and LD information [8,9,10]. SuSiE-RSS is a Bayesian sparse regression framework that models genetic effects as a sum of single-effect components, enabling the identification of multiple causal variants within a locus [10,35,39].

The model outputs posterior inclusion probability (PIP) for each SNP, representing the probability that a variant contributes to the observed association signal [10,11,34]. Although SuSiE-RSS can generate credible sets of candidate causal variants, credible sets were not used for variant prioritization in the present study because the objective was to compare SNP ranking strategies. Instead, all SNPs were ranked according to their posterior inclusion probabilities, and top-ranked SNP subsets were selected for downstream analyses.

### 2.9. SNP Selection, Evaluation, and Genomic Prediction Framework

#### 2.9.1. SNP Prioritization

To systematically evaluate and compare SNP prioritization strategies, an integrated analytical framework was established, encompassing SNP selection, explanatory power assessment, and genomic prediction, facilitating the identification of robust and biologically meaningful candidate variants [1,3,8].

First, SNP subsets were constructed based on two alternative prioritization strategies: (i) GWAS-based ranking using *p*-values and (ii) fine-mapping-based ranking using posterior inclusion probabilities (PIPs) derived from SuSiE-RSS [1,10]. For each method, SNPs were sorted according to their statistical importance, and top-ranked subsets (e.g., top 100, 200, 300, etc.) were selected for downstream analyses.

#### 2.9.2. Explanatory Power Assessment

Second, the explanatory power of selected SNPs was assessed at both individual and joint levels [4,34]. For single-SNP evaluation, each variant was independently fitted using a linear regression model, and the coefficient of determination (R^2^) was used to quantify the proportion of phenotypic variance explained [1]. For multi-SNP evaluation, SNPs were incrementally incorporated into regression models according to their ranking [32,38]. To account for multicollinearity induced by linkage disequilibrium, ridge regression was applied, and the cumulative explanatory power was evaluated using R^2^ across increasing SNP set sizes.

#### 2.9.3. Genomic Prediction

Finally, the predictive performance of selected SNP subsets was evaluated using genomic prediction models [40]. For each SNP subset, a genomic relationship matrix (GRM) was constructed following the standard approach based on marker genotypes, capturing the realized genetic similarity among individuals [41].

Genomic best linear unbiased prediction (GBLUP) models were fitted using the sommer package (v4.4.3) in R [40,41]. In this framework, SNP effects are modeled as random effects under the assumption of a normally distributed genetic architecture, and the GRM serves as the covariance structure among individuals.

To evaluate the impact of SNP prioritization strategies on prediction performance, separate GRMs were constructed from SNP subsets derived from GWAS ranking or SuSiE-RSS fine-mapping results. Prediction performance was then compared across different SNP subsets and selection strategies.

Prediction performance was evaluated using 10-fold cross-validation. Individuals were randomly divided into ten approximately equal subsets. In each iteration, nine subsets were used as the training population, and the remaining subset was used for validation. This procedure was repeated until each subset had served once as the validation set.

Predictive metrics were calculated for each validation fold and subsequently averaged across folds. The corresponding standard deviations were used to assess prediction stability across cross-validation replicates.

For continuous traits, prediction accuracy was assessed using Pearson’s correlation coefficient (PCC) and mean squared error (MSE) [1]. For binary traits, predictive performance was evaluated using the area under the receiver operating characteristic curve (AUC) and Brier score [31,34].

### 2.10. Functional Annotation and Enrichment Analysis

To investigate the biological relevance of candidate variants, functional annotation and enrichment analyses were performed on SNPs prioritized from SuSiE-RSS fine-mapping results. For each trait, SNPs were ranked according to their PIPs [10]. The top-ranked SNPs were then mapped to genes based on their genomic coordinates using the Sus scrofa reference genome annotation (Sscrofa11.1), with gene annotation obtained from the corresponding GFF file (genomic.gff) [13,33]. SNPs located within gene regions were assigned to the corresponding genes, whereas intergenic SNPs were assigned to the nearest gene based on physical distance. Gene annotation parsing and SNP-to-gene mapping were performed using custom Python scripts, which are available from the corresponding author upon reasonable request. When multiple SNPs were mapped to the same gene, each gene was retained only once to avoid redundancy [9]. The top 500 unique candidate genes for each trait were selected for downstream analysis.

Gene Ontology (GO) and Kyoto Encyclopedia of Genes and Genomes (KEGG) pathway enrichment analyses were conducted using the clusterProfiler package (v4.10.0) in R to characterize the functional roles of candidate genes [42,43]. Enrichment analysis was performed using the hypergeometric test with the set of all annotated genes in the corresponding species as the background [35,44]. Multiple testing correction was carried out using the Benjamini–Hochberg (BH) procedure, and terms with a false discovery rate (FDR) < 0.05 were considered significant [34]. The enrichment results were visualized using bubble plots generated with ggplot2, highlighting both shared and trait-specific biological processes and pathways, thereby providing insights into the molecular mechanisms underlying complex traits [44].

Custom Python scripts (Python v3.9) were used for data preprocessing, SNP prioritization, and SNP-to-gene mapping. Statistical analyses, fine-mapping, genomic prediction, and enrichment analyses were performed in R (v4.4.3) using the corresponding packages described above.

## 3. Results

### 3.1. Data Quality Control and Population Structure

The WGS dataset included 451 pigs and 9,600,601 variants. After stringent quality control procedures, all individuals were retained, and 9,512,810 high-quality variants remained. For reference panel construction, additional variant standardization procedures were applied. After restricting the dataset to biallelic SNPs, 7,926,862 variants were retained. Subsequently, strand-ambiguous SNPs (A/T and C/G) were removed, resulting in a final set of 6,909,928 variants for haplotype phasing and reference panel construction.

**Figure 1:**
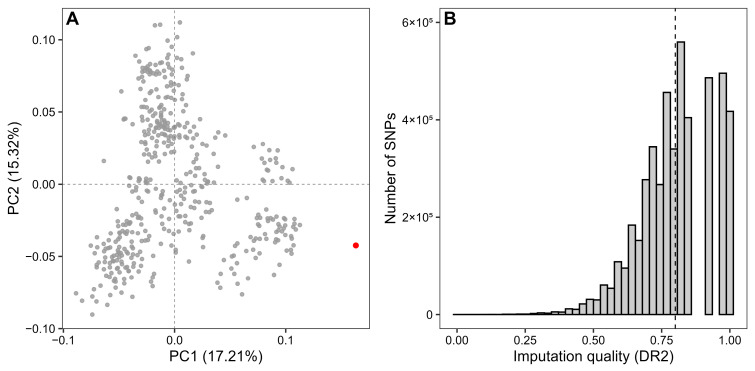
Quality control and genotype imputation: (**A**) PCA based on LD-pruned variants. Grey points represent individuals retained for downstream analyses, whereas the red point indicates the individual identified as a population outlier (Z-score > 3) and subsequently removed. (**B**) Distribution of imputation quality (DR^2^) across all variants. The dashed vertical line indicates the commonly used threshold of 0.8.

Population structure was assessed using PCA based on LD-pruned variants. The first two principal components explained 17.21% and 15.32% of the total genetic variance, respectively (Figure 1A). The PCA revealed a relatively homogeneous population structure, with no apparent clustering pattern. One potential outlier individual was identified based on a Z-score threshold of 3 derived from the leading principal components and was subsequently removed.

The remaining 450 WGS individuals were used to construct the reference haplotype panel for genotype imputation. A separate chip-genotyped population consisting of 320 individuals was subsequently imputed using this reference panel. Alignment between the chip dataset and the reference panel identified 63,742 overlapping SNPs, which were retained as anchor markers for downstream genotype imputation.

Genotype imputation demonstrated high overall accuracy. The distribution of imputation quality (DR^2^) values showed a mean of 0.834 and a median of 0.86, with the majority of variants exceeding the commonly used threshold of 0.8 (Figure 1B). After filtering, a total of 4,443,258 high-confidence SNPs were retained. After imputation quality control, the imputed chip dataset (*n* = 320) was merged with the WGS reference dataset (*n* = 450), resulting in a final dataset of 770 individuals and 4,443,258 SNPs for downstream analyses.

The final merged dataset was subsequently used for GWAS, fine-mapping, and genomic prediction analyses.

### 3.2. GWAS and Fine-Mapping for Teat Traits

**Figure A1:**
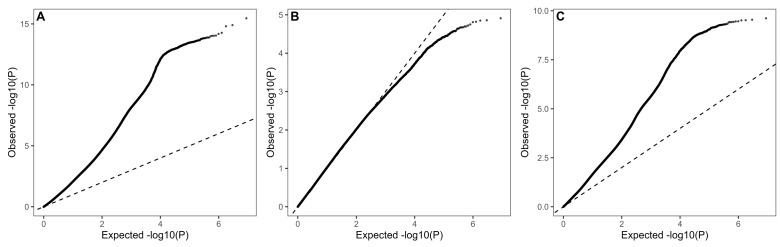
Quantile–quantile (QQ) plots of GWAS results: (**A**) total teat number, (**B**) teat symmetry, and (**C**) teat adequacy. The diagonal dashed line represents the null expectation under no association.

Across the three investigated teat-related traits, GWAS revealed marked heterogeneity in genetic signal strength (Supplementary Data S1). The overall distribution of association statistics, as assessed by quantile–quantile (QQ) plots, indicated substantial differences in signal enrichment and genomic inflation across traits (Figure A1).

**Figure 2:**
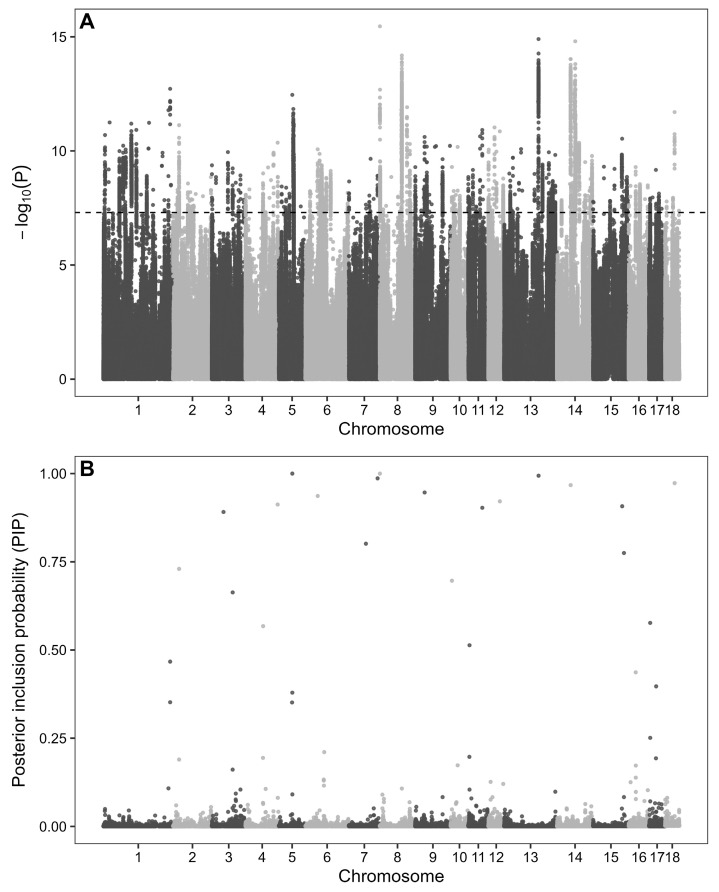
GWAS and fine-mapping for TTN: (**A**) Manhattan plot of GWAS. The dashed horizontal line indicates the conventional genome-wide significance threshold (*p* = 5 × 10^−8^). (**B**) Manhattan plot of PIPs derived from SuSiE-RSS.

For TTN, a strong enrichment of association signals was observed, with a large number of variants reaching genome-wide significance and a substantial elevation in the genomic inflation factor (λGC = 2.50; Figure A1A). Fine-mapping further refined these signals, identifying multiple high-confidence variants with large PIPs, including those approaching or equal to 1, indicating well-resolved signals at specific loci (Figure 2A,B; Supplementary Data S2).

**Figure A2:**
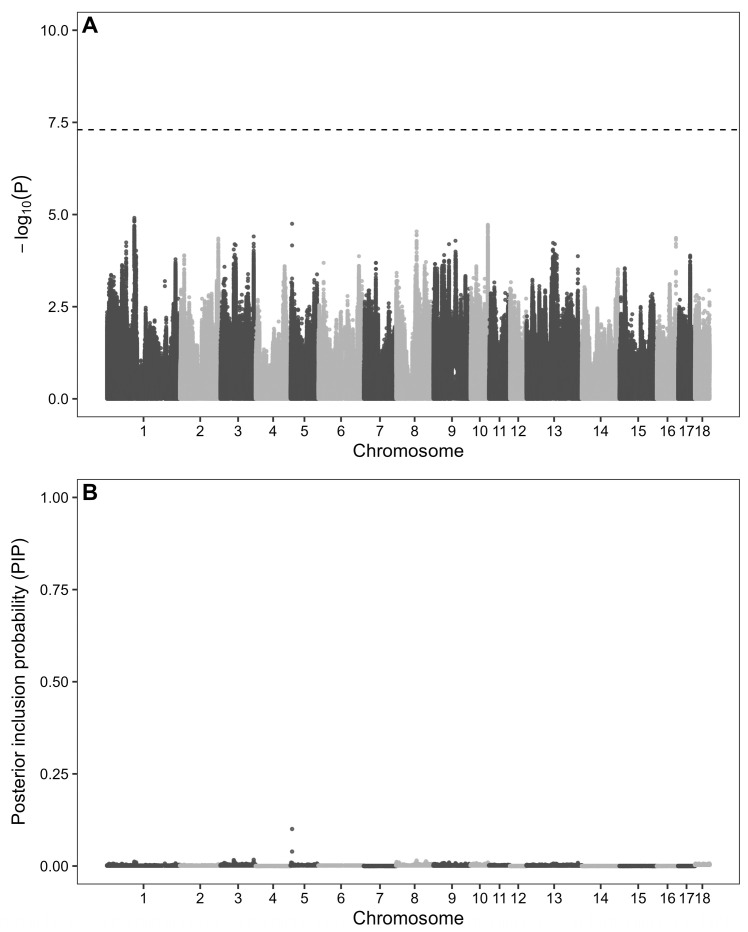
GWAS and fine-mapping for teat symmetry: (**A**) Manhattan plot of GWAS. (**B**) Manhattan plot of posterior inclusion probabilities (PIPs). Genomic positions are aligned between the two panels.

**Figure A3:**
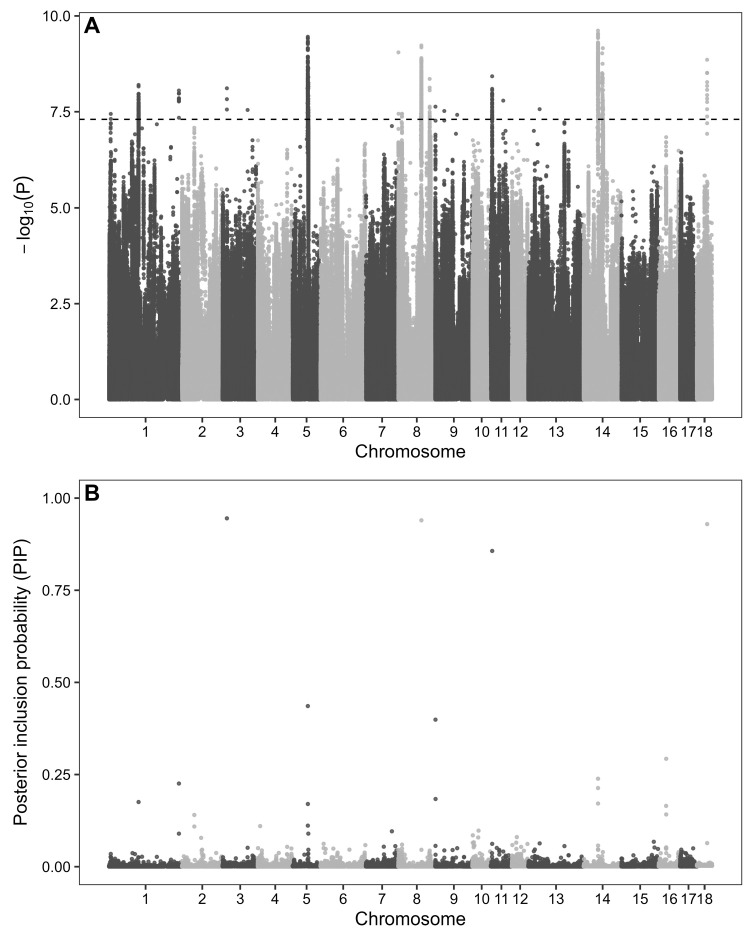
GWAS and fine-mapping for teat adequacy: (**A**) Manhattan plot of GWAS. (**B**) Manhattan plot of posterior inclusion probabilities (PIPs).

In contrast, TS exhibited weak association signals, with no variants surpassing genome-wide significance and genomic inflation factors close to unity (λGC = 1.01; Figure A1B). Consistently, fine-mapping yielded only a very small number of low-confidence candidate variants (Figure A2). For TA, a moderate number of genome-wide significant variants was detected, with a noticeable but less pronounced level of genomic inflation (Figure A1C). Fine-mapping identified a limited set of high-confidence variants (λGC = 1.91; Figure A3).

Thus, the number and strength of detectable association signals varied substantially across traits, ranging from strong signal enrichment to weak or nearly undetectable association patterns. Importantly, fine-mapping provides an additional layer of resolution by refining association signals and prioritizing likely causal variants.

### 3.3. SNP Prioritization on TTN

To enable quantitative assessment of the explanatory power of single-SNP and multiple SNPs, analyses were conducted using the continuous trait (TTN) as an example, since the coefficient of determination (R^2^) is not directly applicable to binary traits.

#### 3.3.1. Single-SNP Explanatory Power

**Figure 3:**
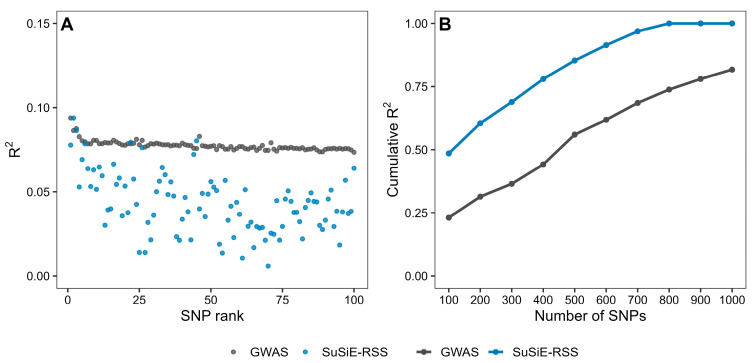
Comparative evaluation of SNP prioritization strategies using single-SNP and multi-SNP explanatory power: (**A**) Single-SNP explanatory power (R^2^) of the top 100 variants ranked by GWAS *p*-values and SuSiE-RSS PIPs. (**B**) Cumulative explanatory power (R^2^) as a function of the number of included SNPs.

As shown in Figure 3A, the top-ranked GWAS SNP achieved a slightly higher explanatory power (R^2^ = 0.0938) compared to the top-ranked SuSiE-RSS SNP (R^2^ = 0.0777). Across the top 100 SNPs, GWAS consistently exhibited higher per-SNP explanatory power, with a mean R^2^ of 0.0774, whereas it was lower for SuSiE-RSS-ranked SNPs (0.0432). A similar pattern was observed for the median R^2^ values (0.0770 for GWAS vs. 0.0420 for SuSiE-RSS).

#### 3.3.2. Multi-SNP Joint Explanatory Power

The cumulative explanatory power of SNP sets by incrementally incorporating top-ranked variants into multi-SNP models was assessed.

In contrast to the single-SNP results, SuSiE-RSS-based SNP sets demonstrated improved performance when evaluated jointly (Figure 3B). Using the top 1000 SNPs, the SuSiE-RSS-based model achieved a cumulative R^2^ of 1.0, indicating near-complete explanation of phenotypic variance in the training dataset and exceeding that of the GWAS-based model (R^2^ = 0.8170), corresponding to an absolute improvement of 0.1830 and a relative increase of 22.4%. Furthermore, the growth trajectory of cumulative R^2^ indicated a steeper increase for SuSiE-RSS compared to GWAS, suggesting that SuSiE-RSS-prioritized SNPs captured complementary genetic signals more effectively as additional variants are included.

Taken together, a fundamental distinction exists between SNP prioritization strategies. While GWAS ranking favors variants with strong marginal effects, it is susceptible to redundancy due to LD, leading to diminishing returns in multi-SNP settings. In contrast, SuSiE-RSS reduces redundancy by identifying multiple independent signals, thereby improving joint explanatory power.

### 3.4. Genomic Prediction

To evaluate the practical utility of SNP prioritization strategies, genomic prediction performance was assessed using SNP subsets ranked by GWAS *p*-values and SuSiE-RSS PIPs (Supplementary Data S3). For each method, genomic relationship matrices (GRMs) were constructed from progressively larger SNP subsets (100–1000 variants), and prediction accuracy was evaluated using appropriate metrics for continuous and binary traits.

**Figure 4:**
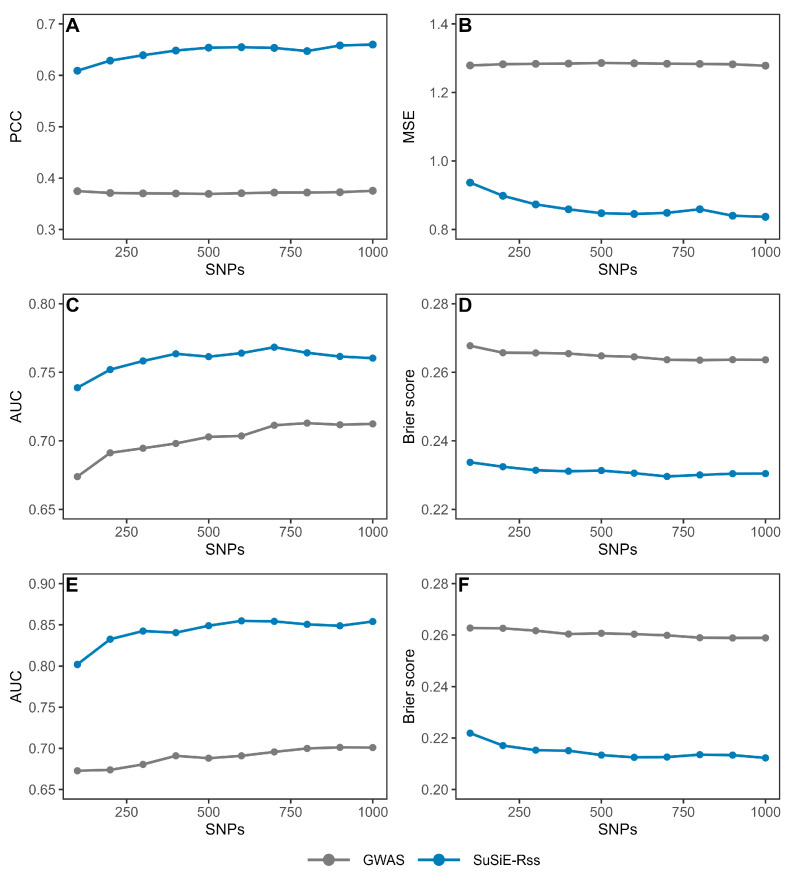
Genomic prediction performance based on SNP prioritization strategies across continuous and binary traits. GBLUP for the continuous trait (TTN): (**A**) PCC; (**B**) MSE. GBLUP for binary traits (TS and TA): (**C**,**E**) AUC; (**D**,**F**) Brier score.

For TTN, prediction performance was evaluated using GBLUP models (Figure 4A,B). Across all SNP subset sizes, SNPs prioritized by SuSiE-RSS consistently outperformed those selected by GWAS, reflected by higher PCC and lower MSE. Notably, the best performance for SuSiE-RSS was achieved with the largest SNP set (1000 SNPs), yielding a PCC of 0.6599 and an MSE of 0.8372, compared to 0.3755 (PCC) and 1.2782 (MSE) for GWAS. Moreover, the performance gain of SuSiE-RSS became evident even at relatively small SNP set sizes and remained stable as additional variants were included. In contrast, GWAS-based prediction showed only marginal improvement with increasing SNP numbers, suggesting limited efficiency in capturing additional informative signals.

For binary traits (TS and TA), prediction performance was evaluated using GBLUP models (Figure 4C–F). Consistent with the results observed for the continuous trait, SNP subsets derived from SuSiE-RSS achieved systematically higher AUC and lower Brier scores compared to GWAS-based SNP selection. For TS, the highest AUC achieved by SuSiE-RSS was 0.7683, compared to 0.7129 for GWAS. Similarly, for TA, SuSiE-RSS reached a peak AUC of 0.8547, substantially higher than the GWAS-based maximum of 0.7012. Across both traits, SuSiE-RSS also consistently produced lower Brier scores, indicating improved calibration of predicted probabilities. The performance gap between the two strategies was most pronounced at intermediate SNP set sizes and tended to stabilize as more variants were included.

Across both continuous and binary traits, a consistent pattern emerged: while GWAS-based SNP selection prioritizes variants with strong marginal effects, it may introduce redundancy due to LD, leading to diminishing returns in predictive performance. In contrast, SuSiE-RSS prioritization, by leveraging fine-mapping information, enables more efficient capture of complementary genetic signals, thereby improving the efficiency of GRM construction and predictive performance.

### 3.5. Functional Enrichment Reveals Trait-Specific Biological Programs

**Figure 5:**
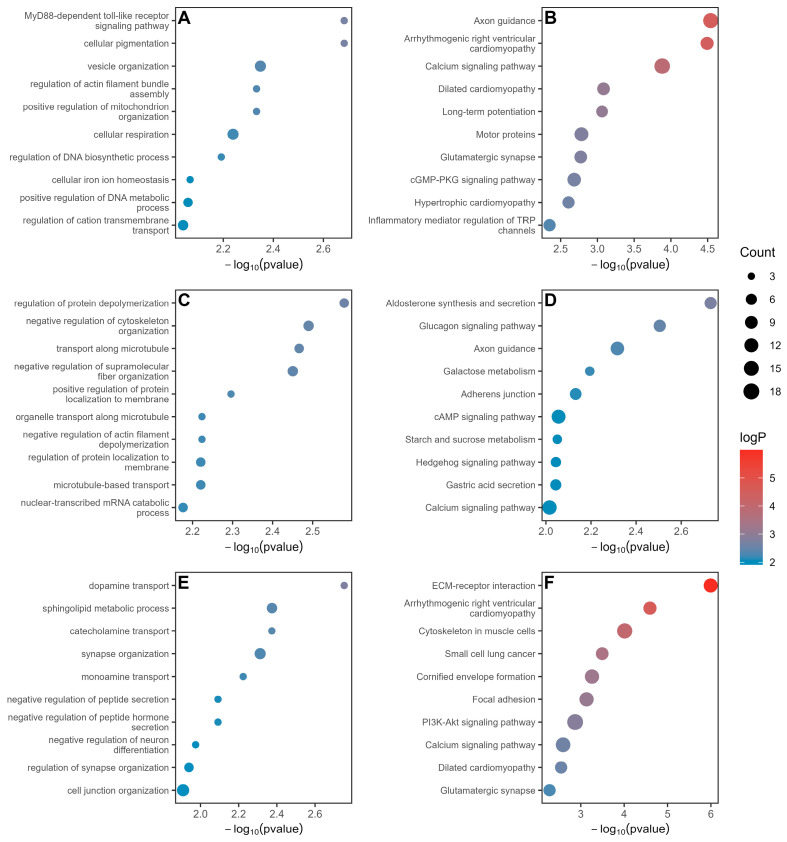
Functional enrichment of candidate genes prioritized by SuSiE-RSS across teat-related traits. Top enriched Gene Ontology (GO) biological processes (**A**,**C**,**E**) and KEGG pathways (**B**,**D**,**F**) are presented for TTN (**A**,**B**), TS (**C**,**D**), and TA (**E**,**F**). Candidate genes were derived from SuSiE-RSS fine-mapping results and ranked based on enrichment significance, with the top 10 terms displayed for each trait. The x-axis represents enrichment significance (−log10(*p*-value)), and point size corresponds to the number of genes associated with each term.

Broadly, these traits exhibited clearly divergent enrichment patterns, with TTN primarily associated with developmental and signaling processes, TS linked to cytoskeletal organization and spatial regulation, and TA enriched in pathways related to cellular interactions and tissue integrity (Figure 5).

For TTN, enrichment analysis found neuro-developmental processes and cellular morphogenesis, with key pathways including axon guidance, calcium signaling, and glutamatergic synapse. Representative candidate genes such as WT1, TBX2, and PPARGC1A are involved in developmental regulation and cellular differentiation, while ABL1 plays an important role in signal transduction and cell proliferation. In addition, NOX4, SLC39A8, and HMGB1 contribute to cellular signaling and stress response processes.

For TS, the most prominent signals were related to tissue remodeling and spatial organization, with representative pathways including axon guidance, Hedgehog signaling, and adherens junction. Candidate genes such as ITGB1, RHOQ, and LIMA1 are directly involved in cell adhesion, cytoskeletal organization, and polarity establishment, while MAP1B, KIF24, and KTN1 participate in microtubule dynamics and intracellular transport.

For TA, enrichment analysis highlighted pathways involved in structural integrity and cellular interactions, particularly ECM–receptor interaction, focal adhesion, and PI3K–Akt signaling. Key candidate genes, including CTNNB1, WNT2, GLI2, MTOR, and RBPJ, are central regulators of developmental signaling pathways controlling cell proliferation, differentiation, and tissue development, while PBX1 and ACVR1C contribute to tissue homeostasis.

Notably, PRKN and AGK were consistently identified among the top fine-mapped candidate genes across all three traits following SNP-to-gene mapping, suggesting potential pleiotropic roles in regulating fundamental cellular processes.

Overall, these results demonstrate a clear functional partitioning among traits: TTN is driven by developmental patterning, TS by spatial organization, and TA by structural integrity and functional stability, with distinct yet partially overlapping genetic components contributing to each trait.

## 4. Discussion

Our results showed that SNP prioritization from purely whole-genome significance-based ranking did not help with the genetic dissection of complex traits [1,2,3,8,9]. By integrating fine-mapping into SNP selection, accounting for LD structure and multi-signal effects, this framework improves predictive performance while enhancing biological interpretability [40,41]. More broadly, SNP prioritization should be viewed not merely as a ranking problem but as a structured representation of genetic architecture, with important implications for genomic selection and functional genomics.

Previous studies also showed that GWAS-based ranking tends to prioritize variants with strong marginal effects, whereas fine-mapping redistributes statistical support across correlated loci [5,9,10]. Similar patterns have been observed in both human and livestock systems [2,3,9], where Bayesian approaches such as SuSiE, CAVIAR, and FINEMAP improve credible set resolution and reduce LD-driven false prioritization [10,11,12,39]. Extending beyond these studies, the present work evaluates SNP prioritization strategies within a unified framework linking explanatory power, genomic prediction, and biological interpretation, thereby providing a more integrative view of their downstream consequences.

A central observation is the divergence between marginal and joint performance [5,34], reflecting a fundamental distinction between signal amplification in GWAS and signal decomposition in fine-mapping approaches [9,35]. While GWAS-ranked SNPs exhibit stronger single-marker explanatory power [4], this advantage diminishes in multi-SNP settings, likely due to redundancy among correlated variants under polygenic architectures [4,37]. In contrast, fine-mapping-based prioritization, by explicitly modeling LD structure and allowing multiple signals within loci [10,39], more efficiently captures complementary genetic effects and achieves superior joint explanatory performance [9,33].

These differences extend to genomic prediction. Consistent with observations in livestock breeding, incorporating biologically informed SNP prioritization improves predictive accuracy relative to significance-based selection [2,3,40]. The superior performance of SuSiE-RSS-derived SNP sets likely reflects reduced redundancy and improved representation of independent genetic signals [10,37], resulting in the construction of more informative genomic relationship matrices [41]. At the same time, these improvements should be interpreted within the context of study design, as they may depend on factors such as sample size, LD structure, and validation strategy [4,6].

Beyond statistical and predictive performance, SNP prioritization also helps with biological interpretation. GWAS-based enrichment analyses often yield broad or inconsistent functional categories, partly due to the inclusion of correlated, non-causal variants [9,33]. In contrast, fine-mapping-based prioritization produces more coherent and trait-specific enrichment patterns [13,33], suggesting a clearer link between genetic signals and the underlying biological processes [13]. Specifically, total teat number was associated with pathways related to developmental patterning and intercellular signaling, including axon guidance, calcium signaling, and glutamatergic synapse, supporting the role of coordinated morphogenetic processes in determining organ number [45,46,47]. Teat symmetry was enriched for pathways involved in cytoskeletal organization, cell adhesion, and spatial regulation, such as adherens junction and Hedgehog signaling, indicating that this trait is governed by mechanisms controlling cellular polarity and tissue-level organization [48,49]. For teat adequacy, enrichment of extracellular matrix interaction, focal adhesion, and PI3K–Akt signaling pathways highlights the importance of structural integrity, cell–matrix interactions, and regulatory signaling in maintaining functional competence [49,50]. Compared with previous studies, these associations were refined into a more targeted and non-redundant set of candidate variants, improving interpretability [19,20].

The integration of candidate gene information further supports these interpretations. Genes such as WT1, TBX2, and CTNNB1 are well established in developmental regulation [51], whereas ABL1, MAP1B, and RBPJ are involved in signal transduction, cytoskeletal dynamics [52], and Notch-related pathways. Notably, these genes converge on a limited number of biological processes rather than representing independent signals, supporting the functional relevance of fine-mapping-based prioritization. In addition, the consistent identification of genes such as PRKN and AGK across traits suggests potential pleiotropic roles in fundamental cellular processes.

Despite these advantages, several limitations should be acknowledged. The performance of fine-mapping depends on accurate LD estimation and model assumptions and may be sensitive to sample size and population structure [8,9]. Although population structure was assessed using PCA and no obvious stratification was detected, the inclusion of additional covariates such as principal components, litter effects, and other environmental factors may further reduce residual confounding and should be considered in future studies. In addition, the observed deviation of QQ plots from the null expectation for some traits may partly reflect the highly polygenic architecture of teat-related traits, where numerous loci with small-to-moderate effects contribute to phenotypic variation. Therefore, inflation in test statistics may not solely originate from population stratification or technical artifacts but may also be influenced by the accumulation of genuine association signals. Nevertheless, mixed-model GWAS approaches and additional covariate adjustments may further improve control of residual confounding in future studies. Bayesian approaches such as SuSiE rely on prior assumptions that may influence posterior inference [35,39]. Furthermore, gene assignment based on physical proximity does not fully capture long-range regulatory interactions. Importantly, GWAS remains a valuable tool for initial signal detection, particularly in large-scale datasets where computational efficiency is critical [1,4]. Fine-mapping should therefore be viewed as a complementary approach that enhances, rather than replaces, GWAS.

## 5. Conclusions

Fine-mapping-based prioritization provided an efficient framework for representing genetic signals through explicit modeling of LD structure and multiple association signals. Within the analyzed population, this strategy showed improved performance in explanatory power, genomic prediction, and biological interpretability compared with conventional GWAS-based ranking. However, further validation in independent populations will be necessary to assess the generalizability of these findings.

## Data Availability

The data presented in this study are available upon request from the corresponding author.

## Supplementary Materials

The following supporting information can be downloaded at: https://www.mdpi.com/article/10.3390/ani16121855/s1, Supplementary Data S1: GWAS summary statistics for teat-related traits, including SNP identifier, chromosome, genomic position, allele information, and association *p*-values. Supplementary Data S2: Fine-mapping results generated using SuSiE-RSS, including trait names, chromosome numbers, genomic positions, SNP identifiers, and posterior inclusion probabilities (PIPs). Supplementary Data S3: Genomic prediction performance results obtained from 10-fold cross-validation, including mean and standard deviation (mean ± SD) of prediction metrics across SNP prioritization strategies and SNP subset sizes.

## Abbreviations

The following abbreviations are used in this manuscript:

GWAS	Genome-Wide Association Study
LD	Linkage Disequilibrium
SNP	Single-Nucleotide Polymorphism
WGS	Whole-Genome Sequencing
QC	Quality Control
MAF	Minor Allele Frequency
HWE	Hardy–Weinberg Equilibrium
PCA	Principal Component Analysis
PC	Principal Component
VCF	Variant Call Format
DR^2^	Imputation Accuracy Metric (Dosage R-squared)
GRM	Genomic Relationship Matrix
GBLUP	Genomic Best Linear Unbiased Prediction
PIP	Posterior Inclusion Probability
SuSiE-RSS	Sum of Single Effects model using Regression with Summary Statistics
R^2^	Coefficient of Determination
PCC	Pearson Correlation Coefficient
MSE	Mean Squared Error
AUC	Area Under the Curve
GO	Gene Ontology
KEGG	Kyoto Encyclopedia of Genes and Genomes
FDR	False Discovery Rate
BH	Benjamini–Hochberg procedure

## Appendix A

### Appendix A.1

**Table A1:** Summary statistics of teat-related traits (*n* = 770 after PCA-based outlier removal).

Trait	No.	Male	Female	Mean ± SD/Proportion	Min	Max	Category 0 (*n*, %)	Category 1 (*n*, %)
Total teat number	770	587	183	11.501 ± 1.215	8	15	—	—
Teat symmetry	770	587	183	0.425	—	—	443 (57.5%)	327 (42.5%)
Teat adequacy	770	587	183	0.458	—	—	417 (54.2%)	353 (45.8%)

## References

[1] Visscher P.M., Wray N.R., Zhang Q., Sklar P., McCarthy M.I., Brown M.A., Yang J. (2017). 10 years of GWAS discovery: Biology, function, and translation. Am. J. Hum. Genet..

[2] Goddard M.E., Hayes B.J. (2009). Mapping genes for complex traits in domestic animals and their use in breeding programmes. Nat. Rev. Genet..

[3] Hayes B., Goddard M. (2010). Genome-wide association and genomic selection in animal breeding. Genome.

[4] Wray N.R., Yang J., Hayes B.J., Price A.L., Goddard M.E., Visscher P.M. (2013). Pitfalls of predicting complex traits from SNPs. Nat. Rev. Genet..

[5] Yang J., Ferreira T., Morris A.P., Medland S.E., Madden P.A., Heath A.C., Martin N.G., Montgomery G.W., Genetic Investigation of ANthropometric Traits (GIANT) Consortium, DIAbetes Genetics Replication And Meta-Analysis (DIAGRAM) Consortium (2012). Conditional and joint multiple-SNP analysis of GWAS summary statistics identifies additional variants influencing complex traits. Nat. Genet..

[6] Price A.L., Zaitlen N.A., Reich D., Patterson N. (2010). New approaches to population stratification in genome-wide association studies. Nat. Rev. Genet..

[7] Bulik-Sullivan B.K., Loh P.R., Finucane H.K., Ripke S., Yang J., Patterson N., Neale B.M. (2015). LD score regression distinguishes confounding from polygenicity in genome-wide association studies. Nat. Genet..

[8] Li Z., Zhou X. (2025). Towards improved fine-mapping of candidate causal variants. Nat. Rev. Genet..

[9] Schaid D.J., Chen W., Larson N.B. (2018). From genome-wide associations to candidate causal variants by statistical fine-mapping. Nat. Rev. Genet..

[10] Zou Y., Carbonetto P., Wang G., Stephens M. (2022). Fine-mapping from summary data with the sum of single effects model. PLoS Genet..

[11] Benner C., Spencer C.C., Havulinna A.S., Salomaa V., Ripatti S., Pirinen M. (2016). FINEMAP: Efficient variable selection using summary data from genome-wide association studies. Bioinformatics.

[12] Hormozdiari F., Kichaev G., Yang W.Y., Pasaniuc B., Eskin E. (2015). Identification of causal genes for complex traits. Bioinformatics.

[13] Farh K.K.H., Marson A., Zhu J., Kleinewietfeld M., Housley W.J., Beik S., Bernstein B.E. (2015). Genetic and epigenetic fine mapping of causal autoimmune disease variants. Nature.

[14] Rohrer G.A., Nonneman D.J. (2017). Genetic analysis of teat number in pigs reveals developmental pathways independent of vertebra number and loci affecting specific sides. Genet. Sel. Evol..

[15] Lopes M.S., Bastiaansen J.W.M., Harlizius B., Knol E.F., Bovenhuis H. (2014). A genome-wide association study reveals dominance effects on number of teats in pigs. PLoS ONE.

[16] Sell-Kubiak E., Duijvesteijn N., Lopes M.S., Janss L.L.G., Knol E.F., Bijma P., Mulder H.A. (2015). Genome-wide association study reveals novel loci for litter size and its variability in a Large White pig population. BMC Genom..

[17] Hong Y., He X., Wu D., Ye J., Zhang Y., Wu Z., Tan C. (2025). Genome selection and genome-wide association analyses for litter size traits in Large White pigs. Animals.

[18] Zhou J., Fu Y., Zhang Y., Tu W., Huang J., Liang Y., Tan Y. (2026). Genome imputation for genome-wide association study of reproductive traits in Chinese Duroc, Landrace, and Yorkshire pigs: Strategy and validation. Animals.

[19] Ke J., Chen C., Fei J., Luo K., Cheng Y., Yu H., Sun B. (2025). Genome-wide analysis of genetic loci and candidate genes related to teat number traits in Dongliao black pigs. Front. Genet..

[20] Yang Q., Ma F., Yuan J., Zhang Q., Chen Z., Shen Y., Zhou X. (2025). Genome-wide association study identifies QTL and candidate genes associated with teat number in pigs. Anim. Genet..

[21] Anderson C.A., Pettersson F.H., Clarke G.M., Cardon L.R., Morris A.P., Zondervan K.T. (2010). Data quality control in genetic case-control association studies. Nat. Protoc..

[22] Marchini J., Howie B. (2010). Genotype imputation for genome-wide association studies. Nat. Rev. Genet..

[23] Purcell S., Neale B., Todd-Brown K., Thomas L., Ferreira M.A.R., Bender D., Maller J., Sklar P., de Bakker P.I.W., Daly M.J. (2007). PLINK: A tool set for whole-genome association and population-based linkage analyses. Am. J. Hum. Genet..

[24] Patterson N., Price A.L., Reich D. (2006). Population structure and eigenanalysis. PLoS Genet..

[25] Price A.L., Patterson N.J., Plenge R.M., Weinblatt M.E., Shadick N.A., Reich D. (2006). Principal components analysis corrects for stratification in genome-wide association studies. Nat. Genet..

[26] Das S., Forer L., Schönherr S., Sidore C., Locke A.E., Kwong A., Vrieze S.I., Chew E.Y., Levy S., McGue M. (2016). Next-generation genotype imputation service and methods. Nat. Genet..

[27] Browning B.L., Browning S.R. (2016). Genotype imputation with millions of reference samples. Am. J. Hum. Genet..

[28] Browning S.R., Browning B.L. (2011). Haplotype phasing: Existing methods and new developments. Nat. Rev. Genet..

[29] Howie B.N., Donnelly P., Marchini J. (2009). A flexible and accurate genotype imputation method for the next generation of genome-wide association studies. PLoS Genet..

[30] Chang C.C., Chow C.C., Tellier L.C.A.M., Vattikuti S., Purcell S.M., Lee J.J. (2015). Second-generation PLINK: Rising to the challenge of larger and richer datasets. Gigascience.

[31] Loh P.R., Tucker G., Bulik-Sullivan B.K., Vilhjálmsson B.J., Finucane H.K., Salem R.M., Chasman D.I., Ridker P.M., Neale B.M., Berger B. (2015). Efficient Bayesian mixed-model analysis increases association power in large cohorts. Nat. Genet..

[32] Guan Y., Stephens M. (2011). Bayesian variable selection regression for genome-wide association studies and other large-scale problems. Ann. Appl. Stat..

[33] Kichaev G., Pasaniuc B. (2015). Leveraging functional-annotation data in trans-ethnic fine-mapping studies. Am. J. Hum. Genet..

[34] Wakefield J. (2009). Bayes factors for genome-wide association studies: Comparison with P-values. Genet. Epidemiol..

[35] Stephens M., Balding D.J. (2009). Bayesian statistical methods for genetic association studies. Nat. Rev. Genet..

[36] Wallace C. (2021). A more accurate method for colocalisation analysis allowing for multiple causal variants. PLoS Genet..

[37] Vilhjálmsson B.J., Yang J., Finucane H.K., Gusev A., Lindström S., Ripke S., Genovese G., Loh P.-R., Bhatia G., Do R. (2015). Modeling linkage disequilibrium increases accuracy of polygenic risk scores. Am. J. Hum. Genet..

[38] Privé F., Aschard H., Blum M.G.B. (2019). Efficient implementation of penalized regression for genetic risk prediction. Genetics.

[39] Wang G., Sarkar A., Carbonetto P., Stephens M. (2020). A simple new approach to variable selection in regression, with application to genetic fine-mapping. J. R. Stat. Soc. Ser. B.

[40] Meuwissen T.H.E., Hayes B.J., Goddard M. (2001). Prediction of total genetic value using genome-wide dense marker maps. Genetics.

[41] VanRaden P.M. (2008). Efficient methods to compute genomic predictions. J. Dairy Sci..

[42] Ashburner M., Ball C.A., Blake J.A., Botstein D., Butler H., Cherry J.M., Davis A.P., Dolinski K., Dwight S.S., Eppig J.T. (2000). Gene ontology: Tool for the unification of biology. Nat. Genet..

[43] Kanehisa M., Goto S. (2000). KEGG: Kyoto Encyclopedia of Genes and Genomes. Nucleic Acids Res..

[44] Yu G., Wang L.G., Han Y., He Q.Y. (2012). clusterProfiler: An R package for comparing biological themes among gene clusters. OMICS.

[45] Kolodkin A.L., Tessier-Lavigne M. (2011). Mechanisms and molecules of neuronal wiring: A primer. Cold Spring Harb. Perspect. Biol..

[46] Berridge M.J., Bootman M.D., Roderick H.L. (2003). Calcium signalling: Dynamics, homeostasis and remodelling. Nat. Rev. Mol. Cell Biol..

[47] Traynelis S.F., Wollmuth L.P., McBain C.J., Menniti F.S., Vance K.M., Ogden K.K., Hansen K.B., Yuan H., Myers S.J., Dingledine R. (2010). Glutamate receptor ion channels: Structure, regulation, and function. Pharmacol. Rev..

[48] Briscoe J., Thérond P.P. (2013). The mechanisms of Hedgehog signalling and its roles in development and disease. Nat. Rev. Mol. Cell Biol..

[49] Hynes R.O. (2002). Integrins: Bidirectional, allosteric signaling machines. Cell.

[50] Manning B.D., Toker A. (2017). AKT/PKB signaling: Navigating the network. Cell.

[51] Clevers H., Nusse R. (2012). Wnt/β-catenin signaling and disease. Cell.

[52] Kopan R., Ilagan M.X.G. (2009). The canonical Notch signaling pathway: Unfolding the activation mechanism. Cell.

